# Divergence and Convergence of Risky Decision Making Across Prospective Gains and Losses: Preferences and Strategies

**DOI:** 10.3389/fnins.2015.00457

**Published:** 2015-12-16

**Authors:** Yoanna A. Kurnianingsih, O'Dhaniel A. Mullette-Gillman

**Affiliations:** ^1^Department of Psychology, National University of SingaporeSingapore, Singapore; ^2^Neuroscience and Behavioral Disorders Program, Duke-NUS Graduate Medical SchoolSingapore, Singapore; ^3^Singapore Institute for Neurotechnology (SINAPSE), National University of SingaporeSingapore, Singapore

**Keywords:** decision making, gains, losses, risk, preferences, strategy, reflection effect, prospect theory

## Abstract

People choose differently when facing potential gains than when facing potential losses. Clear gross differences in decision making between gains and losses have been empirically demonstrated in numerous studies (e.g., framing effect, risk preference, loss aversion). However, theories maintain that there are strong underlying connections (e.g., reflection effect). We investigated the relationship between gains and losses decision making, examining risk preferences, and choice strategies (the reliance on option information) using a monetary gamble task with interleaved trials. For risk preferences, participants were on average risk averse in the gains domain and risk neutral/seeking in the losses domain. We specifically tested for a theoretically hypothesized correlation between individual risk preferences across the gains and losses domains (the reflection effect), but found no significant relationship in the predicted direction. Interestingly, despite the lack of reflected risk preferences, cross-domain risk preferences were still informative of individual choice behavior. For choice strategies, in both domains participants relied more heavily on the maximizing strategy than the satisficing strategy, with increased reliance on the maximizing strategy in the losses domain. Additionally, while there is no mathematical reliance between the risk preference and strategy metrics, within both domains there were significant relationships between risk preferences and strategies—the more participants relied upon the maximizing strategy the more risk neutral they were (equating value and utility maximization). These results demonstrate the complexity of gains and losses decision making, indicating the apparent contradiction that their underlying cognitive/neural processes are both dissociable and overlapping.

## Introduction

The purpose of decision making is to select the best possible outcome. Broadly, decision making can be divided into two overlapping types—those with potential gains and those with potential losses. While mathematically the sign makes little difference, there is abundant behavioral evidence that quite different cognitive processes may be engaged when outcomes pertain to possible gains vs. possible losses. As a powerful example, simply altering the wording of the same absolute outcome between a relative gain and a relative loss produces stark differences in choices, a phenomenon called the framing effect (Tversky and Kahneman, 1981).

While it is clear that decision making is different between gains and losses, it is unclear what specifically is altered. For example, these differences could be due to alterations in risk preferences or alterations in the information that participants rely upon to make their decisions (their choice strategy).

When faced with probabilistic outcomes (uncertainty), individuals, on average, show differential preferences when choosing between possible gains and possible losses. In prospect theory, individuals are considered on average risk averse for gains (prefer smaller certain rewards to larger uncertain rewards) and risk seeking for losses (prefer a larger possible loss over a smaller certain loss; Kahneman and Tversky, 1979). This pattern of inverted preferences over the gains and losses domains is called the reflection effect, and has been suggested to derive from risk preferences arising from each individual having a common degree of diminishing weight of marginal utility across both gains and losses. This single value (the power function risk preference value) would result in differential behavior across gains and losses, as individuals are drawn toward higher gains and away from higher losses.

It is unclear whether the reflection effect actually occurs within individuals, or is only present when comparing group averages. If present in an individual, then there should be a fixed relationship between risk preferences for gains and risk preferences for losses (negatively correlated, e.g., individuals who are most risk averse for gains should be most risk seeking for losses) and an individual's risk preferences from one domain should be predictive of their risk preference in the other. When empirically tested, the reflection effect has been found in individuals when using hypothetical payoffs (Laury and Holt, 2000), but not with real cash payouts (Cohen et al., 1987; Schoemaker, 1990; Laury and Holt, 2000; Tymula et al., 2013; Kurnianingsih et al., 2015; Mullette-Gillman et al., 2015a,b). It has recently been suggested that the theoretical reflection effect of risk preferences across the gains and losses domains may be the product of studying aggregate behavior and does not exist at the level of individual behavior (Tymula et al., 2013).

The changes in choice behavior between the gains and losses domains may also be due to changes in the strategies individuals employ (what information they use to make their decision). For example, individuals can either attempt to maximize their expected outcomes by fully engaging with the available information, or they may satisfice to reduce the expended effort while sacrificing expected outcomes. The differences in how individuals utilize available information may be influenced by sensitivity toward gains and losses. Loss aversion is a key example of this, in which individuals tend to weight choices more heavily on possible losses (Tversky and Kahneman, 1992), and has been suggested to increase motivation in choice behavior (McCusker and Carnevale, 1995), which can be expressed in the use of a more effortful strategy (requiring more calculation) as they attempt to maximize the expected outcome. Alternatively, Schneider (1992), using hypothetical non-incentivized scenarios, suggested that choices were less consistent when described in a loss frame.

Differential processing of gains and losses is also supported by biological evidence indicating that the underlying neural computations may be separable (for review, see Levin et al., 2012). As examples, (1) amygdala lesions result in impaired decisions for gains but not losses (Weller et al., 2007), (2) numerous brain regions involved in decision making show differential responses to gains and losses—including the orbital frontal cortex, midbrain, ventral striatum, and hippocampus (Elliott et al., 2000; Luking and Barch, 2013), (3) aging results in asymmetric alterations of gains and losses risk preferences (Mikels and Reed, 2009; Weller et al., 2011; Kurnianingsih et al., 2015), (4) sleep deprivation modulates risky decision making strategies for gains, but not for losses (Mullette-Gillman et al., 2015b), and (5) affect manipulations differentially modulate choices across the gains and losses domains (Isen et al., 1988). In contrast, there is also significant evidence of commonalities in the neural regions engaged during gains and losses decision making (for summary see Pessiglione and Delgado, 2015). Interestingly, while multiple studies have suggested that gains and losses value signals may be encoded in the ventromedial prefrontal cortex (vmPFC; Tom et al., 2007; Levy and Glimcher, 2012), a recent meta-analysis of over 200 studies found evidence that the vmPFC may only encode gains (Bartra et al., 2013).

Although such ample evidence shows clear differences between choice behaviors and neural responses in the gains and losses domains, it remains unclear what cognitive processes/neural mechanisms actually drives these differences. To investigate this, we used a monetary gamble task to examine the interrelationships of risk preferences and choice strategies across the gains and losses domains. Critically, we used mirrored and intermixed gains and losses trials, to avoid any potential order or block effects. Our hypotheses were: (1) on average, individuals would be risk averse in the gains domain and risk seeking in the losses domain, (2) individual risk preferences would be uncorrelated across the gains and losses domains, (3) individuals would show higher use of the more effortful and maximizing strategy in the losses domain than in the gains domain. In addition, we examined the predictive power of cross-domain risk preferences on choice behavior and also the interrelationship between risk preferences and choice strategies within and across the gains and losses domains.

## Methods

### Participants

Data was collected from 104 participants (57 females, age *mean* ± *SD* = 23 ± 2.47 years old) that were students from the National University of Singapore. All participants provided written informed consent under a protocol approved by the National University of Singapore Institutional Review Board.

### Monetary gamble task design

Risk preference and choice strategy measures were quantified based on participant's performance on a monetary gamble task. Data collection and analyses were accomplished using MATLAB (Mathworks, Natick, MA) with the Psychophysics Toolbox for trial presentation (Brainard, 1997), and R Statistical Software (R Core Team, 2013).

**Figure 1:**
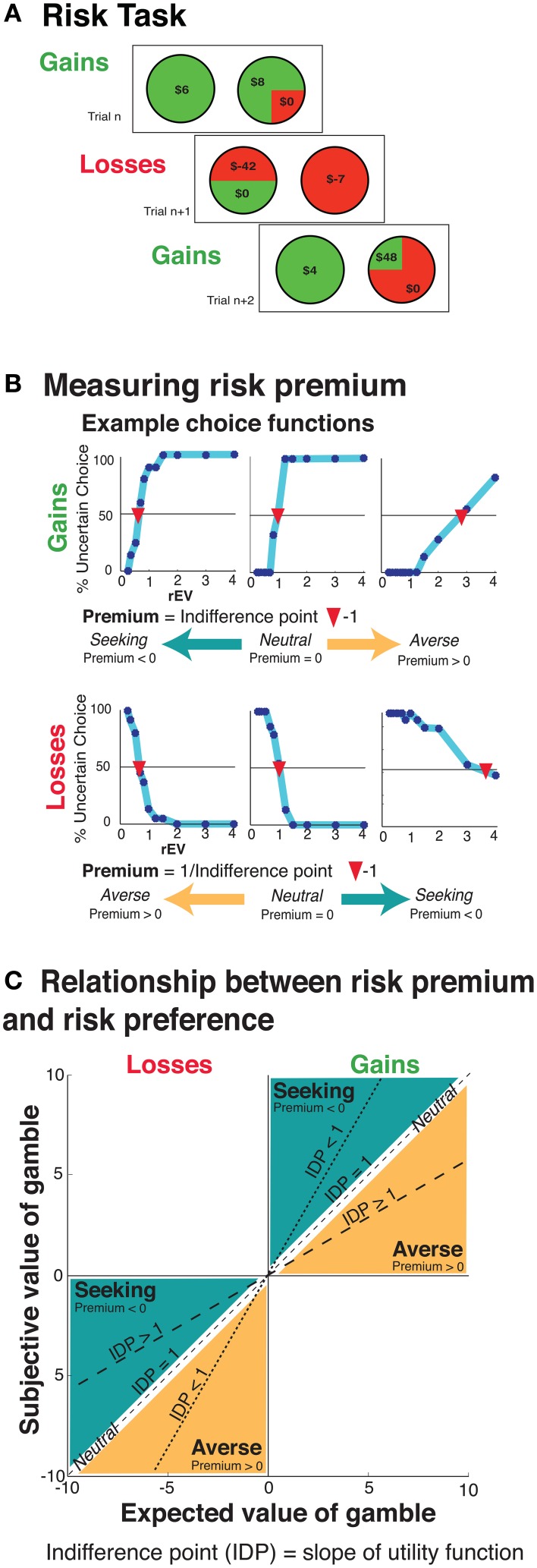
**(A)** Example task trials. In each trial, participants chose between a certain and a risky option. There were two types of trials, the gains and losses trials, randomly intermixed. **(B)** Example individual choice functions for six individuals (top: 3 for gains, bottom: 3 for losses). Choice functions were plotted within each domain for each participant. Each relative expected value (x-axis) was plotted against the percentage of trials (out of 15 for each point) at which the participant selected the risky option (y-axis). **(C)** An illustration describing the relationship between the relative expected value of the gamble (x-axis), the subjective value of the gamble (y-axis) and risk premium (slope of the lines).

The monetary gamble task consisted of 165 gains trials and 165 losses trials (Figure 1A). On each trial, participants chose between a certain option and a gamble option. All gambles featured a possible $0 outcome, to provide a clear and consistent anchor point across all trials, to ensure the frame in which participants considered the possible outcomes (Tversky and Kahneman, 1974). Within the gains trial, there were five different certain gain options ($3, $4, $5, $6, $7). Gain gambles were constructed based upon three probabilities of winning [pWIN, which are (25, 50, 75%)], and 11 different relative expected values [rEV or EV_Gamble_/V_Certain_, which are (0.25, 0.33, 0.50, 0.66, 0.80, 1.0, 1.25, 1.5, 2.0, 3.0, and 4.0)]. These probabilities and relative expected values resulted in potential gains ranging from $1 to $112. The losses trials were constructed using the same method and values, save for mirroring the valence of the values offered into negatives. The trial order was fully randomized separately for each participant, intermixing gains and losses domain trials.

Before performing the task, participants were informed that the amount of their compensation for participation would be between $5 and 25, based upon random selection and resolution of one trial from each domain at the end of the experiment. Participants were also informed that they would be paid a proportion of the total monies that they collected, but were not told what that proportion was. Final payment was determined by paying out 50% for the gains task and 25% for the losses task, with the differential to help ensure positive compensation to the participants. On average, participants received $7.77 (*SD* = *$*4.22; *min* = *$*5.00 and *max* = *$*25.00).

Critically, our design features multiple safeguards to prevent potential order, learning, or framing confounds. To prevent order and/or block effects, gains and losses trials were randomly intermixed. To prevent alterations of preferences and/or strategies due to inter-trial learning, no gambles were resolved until the completion of the experiment. To prevent framing confounds, all gambles feature a $0 outcome to provide a clear common anchor across trials.

### Quantifying risk preferences

Risk preferences are commonly quantified using a variety of methodologies. In economics, risk preference is often conceptualized as the curvature of the value to utility function (a power function) due to diminishing marginal utility, based upon expected utility theory (von Neumann and Morgenstern, 1944), and prospect theory (Kahneman and Tversky, 1979). Risk preference has also been quantified as the degree of variance of the expected value (Markowitz, 1952; Bossaerts and Plott, 2004) measured using the coefficient of variation (CV), which is calculated as the ratio of the standard deviation to the expected value of the gamble (Weber et al., 2004).

In psychology, risk-taking behavior has been examined using a large range of tasks and models, including the Iowa Gambling Task (IGT; Bechara et al., 1994), the Balloon Analogue Risk Task (BART; Lejuez et al., 2002), and the Cambridge Gamble Task (CGT; Rogers et al., 1999). In such tasks, risk preference is often quantified by the proportion of times the participant chooses the riskier option.

As a midpoint, in this study we used a model free psychometric approach to empirically examine risk preferences (Stanton et al., 2011; Kurnianingsih et al., 2015; Mullette-Gillman et al., 2015a,b). This method quantifies risk preference (in each domain) as a risk premium metric, which measures the degree to which people appear to alter the subjective value of gambles due to outcome uncertainty. In addition, to facilitate comparison with studies from economics and test the robustness of our analyses/results, we replicated analyses utilizing the power function metric.

#### Risk premium metric

To quantify this psychometric measure of risk, choice functions were constructed by plotting a continuous function based upon the percentage of gamble selection (y-axis) for each respective examined rEV (EV_Gamble_/V_Certain_; Figure 1B). This identifies the point along the rEV axis at which the participant is indifferent between the certain and gamble options (Stanton et al., 2011; Kurnianingsih et al., 2015; Mullette-Gillman et al., 2015a,b). This indifference point is then converted to a risk premium value. In the gains domain, the risk premium is generated by subtracting 1 from the indifference point value (risk premium = indifference point − 1). In the losses domain, as the rEVs are relative to the absolute values, the risk premium is obtained by first inverting the indifference point value before subtracting 1 (risk premium = 1/indifference point − 1).

The risk premium value measures the degree and direction in which an individual modulates the subjective value of a gamble due to the outcome being unknown (Figure 1C). For example, a risk premium value of 1 indicates that the individual requires a gamble to have an expected value twice that of a certain option in order to find the two options equivalent. In other words, they are subjectively halving the expected utility of the gamble due to uncertainty. For both domains, a zero risk premium value reflects no change in valuation (risk neutral), a positive risk premium value denotes diminished valuation (risk averse), and a negative risk premium value denotes enhanced valuation (risk seeking).

We note that participants presented highly monotonic choice functions, as exemplified by the six presented in Figure 1B. A small number of participants had choice functions that did not cross the 50% uncertain choice, preventing us from calculating their indifference point (gains *N* = 13, losses *N* = 5). Such participants were excluded from analyses of their risk premium values. We note that the majority of such participants did not show extreme risk preferences with monotonic choice functions, but rather demonstrated dependence on simple heuristics (such as always choosing the certain option, or always choosing certain for one probability and gamble for others), resulting in choice functions that were flat across our examined range of rEV values, and placing their behavior outside of our functional definition of risk preference. We note that such behavioral heuristics are well-captured by our strategy analyses.

#### Power function metric

We also independently computed the power function metric of risk preference for each domain (based on Tymula et al., 2013).

$$\begin{array}{l} \text{For gains }(\text{if }V>0):SV=\text{pWIN}\times V^{\text{α}}\\ \text{For losses }(\text{if }V<0):SV=-(1-\text{pWIN})\times {\left(-V\right)}^{\text{α}} \end{array}$$

where *SV* is the subjective value (utility), pWIN is the probability of receiving the better outcome of the gamble, *V* is the potential objective value offered and α is the participant's risk preference value. In the gains domain, α < 1 indicates risk averse preference, α = 1 indicates risk neutral preference, and α > 1 indicates risk seeking preference. In the losses domain, the relationship is inverted, such that, α < 1 indicates risk seeking preference, and α > 1 indicates risk averse preference.

To estimate individual's risk preference, we used maximum likelihood to fit the choice data of each participant with the probability choice function (Tymula et al., 2013):

$$\begin{array}{l} \mathrm{Probability of choosing the gamble option}=\frac{1}{1+e^{-\left({\mathrm{SV}}_{G}-{\mathrm{SV}}_{C}\right)}} \end{array}$$

where *SV*_*c*_ is the subjective value of the certain option and *SV*_*G*_ is the subjective value of the gamble option.

We have previously reported strong correlations (*r* > |0.6|) between our risk premium metric and the power function metric (Stanton et al., 2011; Kurnianingsih et al., 2015; Mullette-Gillman et al., 2015a,b), and find similar results in the current sample (see Results).

### Quantifying choice strategy

Choice strategy measures the influence of trial factors on the choices of each participant, as quantified through the use of linear regressions. Analyses were conducted separately within the gains and losses domains, through independent linear regressions to determine the influence of two factors on the choices of each participant: (1) the relative expected value (rEV) of the options, and (2) the probability of winning (pWIN) the gamble option. The R-squared value of each factor gives us a measure of the proportion of individual's choice variance (across trials within domain) accounted for by each factor. Therefore, a high R-squared value for rEV or pWIN would indicate that choices could be well-accounted for based on that specific trial information (e.g., a participant that accepts all gambles with a 75% chance of winning would have a high pWIN R-squared value, and a participant that accepts gambles with an rEV equal or higher than 1 would have a high rEV R-squared value), whereas a low R-squared value would indicate that choices were more likely based on other factors (or were made randomly). It is important to note that based on task design, the pWIN and rEV trial values are orthogonal to each other (the correlation between trial rEV and trial pWIN across all trials is zero). In addition, we note that while we chose to focus on the rEV factor, in this task this factor is essentially isometric with the difference in expected values [for both gains and losses domains, *r*_(102)_ = 0.99, *p* < 0.0001] and is uncorrelated with pWIN [gains *r*_(102)_ = −0.44, *p* < 0.0001; losses *r*_(102)_ = −0.50, *p* < 0.0001].

Based upon the likely outcomes, participants were considered to be “maximizing” when they relied highly on the rEV information and “satisficing” when they relied highly on the pWIN information. The utilization of the rEV information maximizes average outcomes but requires several layers of cognitive calculation, while focusing on the pWIN information allows the use of simple heuristics requiring less cognitive effort.

### Measuring individual numerical ability

The ability to understand and perform simple mathematical calculations was assessed using an 8-item Numeracy Scale developed by Weller et al. (2013). This assessment was given to the participants after they had completed the gamble task, but before resolution of the payments for the choices were made for the gamble task.

### Measuring behavioral impulsiveness

Behavioral impulsiveness was assessed using The Barratt Impulsiveness Scale (BIS-11, Patton and Stanford, 1995). Impulsivity has been considered as a factor influencing risk-taking behaviors (Zaleskiewicz, 2001; Zuckerman, 2007). The 30-item BIS-11 questionnaire consists of three subscales—cognitive, non-planning, and motor. The sum of the subscale scores provides us with a general measure of individual overall impulsiveness. Participants completed this survey after completion of the gamble task, but before resolution of the payments for the choices were made for the gamble task.

## Results

### Response time

As a first comparison of decision behavior between the gains and losses domains, we compared response times between gains and losses trials (see Table 1 for summary of the results). We found that on average, response time were longer for trials in the losses domain [*mean of individual medians* ± *SD* difference = 0.89 ± 0.54 s, gains = 1.90 ± 0.68 s, losses = 2.79 ± 1.00 s; *t*_(103)_ = 16.58, *p* < 0.0001, Cohen's *d* = 1.04].

**Table 1:** **Comparing economic measures between the gains and losses domain**.

	**Gains domain**	**Losses domain**	**Correlation**		***t*****-test**		***z*****-test**	
	***mean ± SD***	***mean ± SD***	***Coefficient (r)***	***p-value***	***t-score***	***p-value***	***z-score***	***p-value***
Response time (s)^*^	0.901 ± 0.675	2.786 ± 1.003	0.860	<0.0001	16.58	<0.0001		
**RISK PREFERENCE**								
(a) Risk Premium (91, 99)^**^	0.441 ± 0.695	0.059 ± 0.296	0.153	0.156	5.89	<0.0001		
(b) Power Function (96, 101)	0.684 ± 0.217	0.984 ± 0.259	0.084	0.395	8.30	<0.0001		
Correlation between a and b	*r* = −0.648, *p* < 0.0001	*r* = 0.583, *p* < 0.0001					0.71	0.478^***^
**CHOICE STRATEGY**								
(c) rEV R-squared (104, 104)	0.338 ± 0.169	0.383 ± 0.117	0.608	<0.0001	3.41	<0.0001		
(d) pWIN R-squared (104, 104)	0.042 ± 0.061	0.035 ± 0.050	0.242	0.013	1.02	0.310		
Correlation between c and d	*r* = −0.443, *p* < 0.0001	*r* = −0.498, *p* < 0.0001					0.50	0.617
**PREFERENCE** × **STRATEGY**								
(e) Premium × rEV R-squared	*r* = −0.143, *p* = 0.177	*r* = −0.436, *p* < 0.0001					2.19	0.029
(f) Premium × pWIN R-squared	*r* = 0.041, *p* = 0.700	*r* = 0.246, *p* = 0.014					1.42	0.156

*Abbreviations: rEV, relative expected value; pWIN, probability of winning; s, seconds; SD, standard deviation*.

* *For response times, median is provided instead of mean*.

** *Numbers in parentheses indicate the number of participants (Gains, Losses)*.

*** *Given the differential relationships between the premium and power metrics across the gains and losses domains, the sign of the correlation in gains was inverted for comparison (comparing 0.648 to 0.583)*.

### Risk preferences

#### Correlations across risk premium and power metrics

To facilitate cross-analytic approaches, we compared the model-free risk premium metric to the model-based risk preference parameter from the power utility function. We found very high correlations between these two risk preference metrics in both the gains [*r*_(89)_ = −0.65, *p* < 0.0001] and the losses domains [*r*_(96)_ = 0.58, *p* < 0.0001], in concurrence with our prior studies (Stanton et al., 2011; Kurnianingsih et al., 2015; Mullette-Gillman et al., 2015a,b). These high correlations indicate that the risk premium and power function metrics are largely capturing the same variance across participants. To confirm this similarity and the robustness of our results, for each analysis we provide the results using both the risk premium and power function metrics.

#### Average risk preference

**Figure 2:**
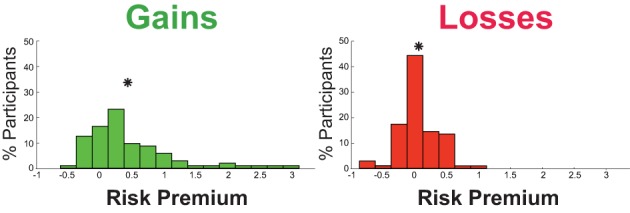
**Risk premium distribution across participants in the gains domain and losses domain**. The asterisk indicates the mean value of each distribution.

Participants were, on average, risk averse in the gains domain [risk premium *mean* ± *SD* = 0.44 ± 0.70; significantly different from 0, *t*_(90)_ = 6.05, *p* < 0.0001, *d* = 0.64] and risk neutral in the losses domain [risk premium *mean* ± *SD* = 0.06 ± 0.30; not significantly different from 0, *t*_(98)_ = 1.97, *p* = 0.052, *d* = 0.20; Figure 2]. Utilizing the power function metric, we replicated the above pattern of results (Table 1). Individuals were on average risk averse for gains [*mean* ± *SD* = 0.68 ± 0.22; significantly different from 0, *t*_(103)_ = 14.84, *p* < 0.0001, *d* = 1.47] and weakly risk seeking for losses [*mean* ± *SD* = 0.95 ± 0.26; significantly different from 0, *t*_(103)_ = 2.06, *p* = 0.04, *d* = 0.20], with Cohen's *d* indicating small effect size for losses (Cohen, 1988).

#### Testing for non-parametric relationships between risk preferences across domains

**Figure 3:**
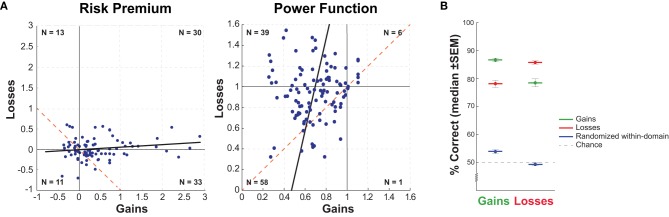
**(A)** Relationship of within-subject risk premium values across the gains and losses domains. The dashed red line visualizes the correlation predicted by the theoretical reflection effect, with a slope of −1 for the risk premium metric (left) and +1 for the power function metric (right). **(B)** Cross-domain predictive comparison, percentage of choice behavior correctly predicted by each risk preference, across both domains. Randomized within-domain power function values were obtained through bootstrap analysis, randomly resampling risk preference value and participant's choice sets independently (*N* = 10,000 iterations, with replacement). The standard error measurement (SEM) value is the median SEM across iterations.

To begin our examination of whether the reflection effect extends from average risk preferences to individual risk preferences, we followed the analysis of Tymula et al. (2013) by conducting a chi-square test to examine if there was evidence of a gross categorical relationship between individual risk preferences for gains and losses. This analysis groups participant based on whether they were risk averse or risk seeking in each domain (Figure 3). Utilizing the risk premium metric, we found no significant relationship across risk preferences across domains [$\chi_{\left(1\right)}^{2}=2.52$, *p* = 0.11], with only 52.9% of the participants showing the pattern of preferences predicted by the reflection effect, indicating no significant relationship between risk preferences across domains. Utilizing the power function metric, 61.5% of the participants had categorical risk preferences in agreement with the reflection effect, which produced significance when examined using the chi-square [$\chi_{\left(1\right)}^{2}=5.51$, *p* = 0.019]. Combined, these results show that the reflection effect is only weakly able to provide categorical prediction of risk preferences.

#### Testing for parametric relationship of risk preferences across domains

The reflection effect predicts a negative correlation between gains and losses risk premiums and a positive correlation between gains and losses power function (Figure 3). We found non-significant positively-signed correlations between the gains and losses domains, for both the risk premium [*r*_(85)_ = 0.15, *p* = 0.16] and power function metrics [*r*_(102)_ = 0.08, *p* = 0.39]. This clearly indicates that the reflection effect is not detectable at the individual level. We note that, in concurrence with the finding of Tymula et al. (2013), we find effects in the opposite direction to that predicted by the reflection effect for the risk premium metric.

#### Modeling risk preferences across domains

To empirically identify the relationships between individual risk preferences across the gains and losses domains, we utilized linear regressions to identify the strength and direction of the predictive relationship.

For the risk premium metric, the reflection effect predicts that the losses risk premium value of each participant can be predicted based upon a transform of the gains risk premium value of that participant:

$$\begin{array}{l} \mathrm{Predicted losses risk premium}=1∕\left(\mathrm{gainsriskpremium}+1\right)-1. \end{array}$$

This is a slightly non-linear transform, which will result in the expected relationship between the gains risk premium and the transformed gains risk premium being dependent upon the distribution under examination. In the current sample, the reflection effect predicts a correlation of −0.85. Using a linear regression to test for the presence of this relationship, we found a non-significant regression equation (*B*_Intercept_ = −0.005, *p* = 0.88; *B*_Slope_ = 0.055, *p* = 0.16) with an R-squared value of 0.023, indicating no evidence for the presence of an individual reflection effect using the risk premium metric.

For the risk power function metric, the reflection effect predicts that losses risk power function values should be equal to gains risk power function values (*B*_Slope_ = 1). Using linear regression yielded a solved equation with a significant intercept, but a non-significant slope (*B*_Intercept_ = 0.88, *p* < 0.0001; *B*_Slope_ = 0.10, *p* = 0.40) with an R-squared value of 0.007. This does not agree with the theoretical reflection effect, as the significance found for the intercept suggests that alterations in risk preferences across the gains and losses domains are due to additive effects, while the theoretical reflection effect is multiplicative. Finally, the very low overall R-squared value indicates the lack of a meaningful predictive relationship between the gain and loss values.

In summary, using both the risk premium and risk power function metrics, we find no evidence for the presence of an individual reflection effect—gains risk preferences cannot predict losses risk preferences.

#### Empirically testing the predictive ability of cross-domain preferences

Without a significant within-subject relationship between risk preferences across the gains and losses domains, an important question is whether there is any predictive power through which cross-domain preferences can predict individual choice behavior. To test this, we calculated the within-subject proportion of choices that were predicted by each domains preference for each domains choices (2 × 2; gains preference predicting gains choices, gains preference predicting losses choices, losses preference predicting gains choices, and losses preference predicting losses choices). The cross-domain prediction measures the proportion of choices predicted by cross-domain preference assuming the reflection effect was true. We chose to limit this analysis to the power function metric, given multiple comparison concerns and the slight non-linearity across the gains and losses domains in the risk premium metric.

To guide interpretation, we determined two reference values for comparison. The first reference was chance, which definitionally is set at 50% (given the two-alternative choice task used). The second reference value accounted for behavioral regularities across participants engaged in this task, by examining scrambling the relationship between individual preferences and individual choices.

This second reference value, the randomized within-domain, was computed (for each domain) based on overall subject behavior in this domain, but with the specific removal of the relationship between individual preferences and individual choice behaviors. In other words, this reference value reflects that there may be regularities in the choice behavior across participants in this task, such that knowledge of any participant's choices may facilitate prediction of another participant's choices (bootstrapped chance). To accomplish this, we ran two bootstrap analyses of 10,000 iterations (one for gains, one for losses). In each iteration, we constructed a new sample of participants (*N* = 104), with replacement, by randomly selecting a preference value and then, independently, randomly selecting a choice set. Doing so specifically breaks the relationship between individual preferences and the actual choice behaviors. For each constructed participant, we then determined the proportion of choices in the randomly-selected choice set that could be predicted by the randomly-selected preference value. For each iteration, we took the mean proportion of choices correctly predicted across that sample. The median proportion across samples (*N* = 10,000), is our second reference value, the randomized within-domain—the proportion of trials that can be expected to be predictable based on knowledge of how participants behave (on average) in the task without specific knowledge of the participants preference value (essentially, removing all within-subject information). In the gains domain, the median value was 53.94% (*SD* = 8.86%) and in the losses domain the median value was 49.39% (*SD* = 6.03%). Comparing the two different reference values, in the gains domain the second reference was slightly higher than chance [*t*_(103)_ = 6.45, *p* < 0.0001, *d* = 0.63], while in the losses domain, there was no significant difference between the two reference values [*t*_(103)_ = 0.67, *p* = 0.50, *d* = 0.07].

The purpose of this analysis was to directly test how well individual preference values are able to predict behavior cross-domain (see Figure 3B). Within the gains domain, an individual's gains risk preference could account for a median of 86.67% (*SD* = 6.67%) of their choice behavior. Within losses, an individual's losses risk preference could account for a median of 85.76% (*SD* = 5.80%) of their choice. Across domains, we see that an individual's risk preference for gains accounts for a median of 78.48% (*SD* = 14.54%) of their choice behavior in the losses domain. Similarly, an individual's risk preference for losses accounts for a median of 78.18% (*SD* = 12.76%) of their choice behavior in the gains domain.

Contrasting these values, we see that a participant's within-domain risk preferences are always better predictors of their choices compared to their cross-domain risk preferences [see Table 2; paired-comparison *t*-tests Gains: *t*_(103)_ = 0.87, *p* < 0.0001, *d* = 0.96; Losses: *t*_(103)_ = 7.59, *p* < 0.0001, *d* = 0.92]. Interestingly, cross-domain risk preferences are still able to predict participant's choices significantly better than the two reference values (chance and bootstrapped chance, all one-sample *t*-tests *t* > 10.88, *p* < 0.0001).

**Table 2:** **Proportion of choices correctly predicted by each domain preference and reference in each domain**.

	**Gains domain**		**Losses domain**		**Across domain *t*-test**	
	***mean%** ± **SD%***		***mean%** ± **SD%***		***t-score***	***p-value***
(a) Gains preference (104)^*^	85.20 ± 6.67		75.02 ± 14.54		7.53	<0.0001
(b) Losses preference (104)	75.49 ± 12.76		85.19 ± 5.80		7.59	<0.0001
(c) Randomized within-domain preference^**^	55.60 ± 8.86		49.60 ± 6.03		>100	<0.0001
	*median* = 53.94		*median* = 49.39			
**Within domain *t*-test**	***t-score***	***p-value***	***t-score***	***p-value***		
a and b	7.53	<0.0001	7.59	<0.0001		
a and c	45.28	<0.0001	15.91	<0.0001		
a and 50% chance	53.83	<0.0001				
b and c	17.84	<0.0001	62.59	<0.0001		
b and 50% chance	6.45	<0.0001	61.87	<0.0001		
c and 50% chance			0.677	0.500		

* *Number in parentheses indicates the number of participants*.

** *The relationship between individual preference and choice behavior was removed, and new samples (each N = 104, with replacement) were reconstructed through random selection of risk preference value and independent random selection of choice set (bootstrap analysis, with N = 10,000 iterations). The values of the bootstrap analysis stated above are the median of the mean and the median of the standard deviation from the 10,000 iterations*.

### Choice strategy

**Figure 4:**
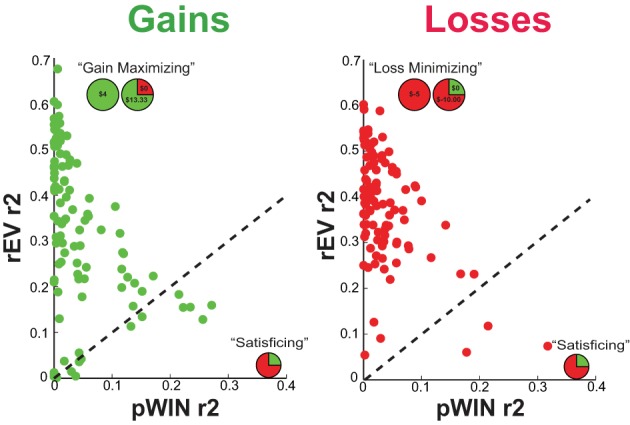
**Choice strategy metric showing the relationship between the amount of trial relative expected value information and trial probability of winning information utilized**. The R-squared value quantifies the amount of choice variances that can be independently explained by each trial factor, relative expected value (rEV) and probability of winning (pWIN).

To examine which information each participant used to make their choices, we quantified the degree to which each participant relied on the trial rEV and pWIN information, as the amount of choice variance that could be explained by each factor (Figure 4). In the gains domain, participants relied more on the use of rEV information (rEV R-squared *mean* ± *SD* = 0.34 ± 0.17) than pWIN information [pWIN R-squared *mean* ± *SD* = 0.04 ± 0.06; *t*_(103)_ = 14.82, *p* < 0.0001, *d* = 2.34], with a negative relationship between the amount of rEV and pWIN information used [*r*_(102)_ = −0.44, *p* < 0.0001]. A similar pattern was found in the losses domain, with higher reliance on rEV information [rEV R-squared *mean* ± *SD* = 0.38 ± 0.12, pWIN R-squared *mean* ± *SD* = 0.04 ± 0.05; *t*_(103)_ = 23.81, *p* < 0.0001, *d* = 3.87] and a negative relationship between the amount of rEV and pWIN information used [*r*_(102)_ = −0.50, *p* < 0.0001].

Across domains, participants relied more on rEV information in the losses domain [*t*_(103)_ = 3.41, *p* < 0.0001, *d* = 0.31], while there was no difference in the amount of pWIN information used [*t*_(103)_ = 1.02, *p* < 0.0001, *d* = 0.12]. Across domains, there were significant correlations in the amount of rEV and pWIN information participants used [rEV: *r*_(102)_ = 0.61, *p* < 0.0001; pWIN: *r*_(102)_ = 0.24, *p* = 0.013].

### Relationship between risk premium and choice strategies

**Figure 5:**
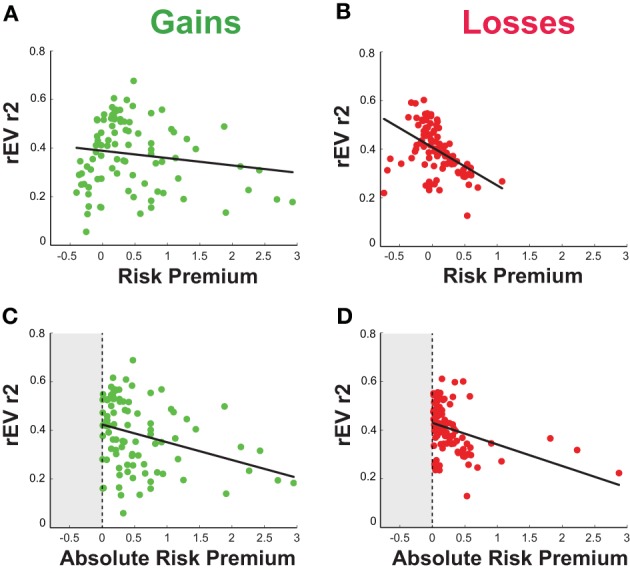
**Relationship between individual risk premium and the degree to which participants relied upon the relative expected value (rEV) information in their choices in the (A) gains and (B) losses domains**. A significant negative correlation is present for losses. Relationship between individual deviation from neutral risk preference (absolute risk premium accounting for the non-linearity across zero) and reliance upon the rEV information in the **(C)** gains and **(D)** losses domains. The vertical dashed line is drawn at risk neutrality (premium = 0), and the now-unattainable negative region is shaded gray. Following this transform, significant negative correlations are seen in both domains, indicating that as participants relied more heavily on the rEV information, their risk preferences became more risk neutral.

We investigated the relationship between the risk preferences and choice strategies within the gains and losses domains (Figures 5A,B; Table 1). We opted to limit these analyses to the risk premium metric, due to multiple comparison concerns, the high correlations between the premium and power function metrics, and our prior use of the premium to examine this issue in a study with blocked trials (Kurnianingsih et al., 2015). Within each domain, we looked separately for correlations between the risk premium and the two choice strategy components, rEV R-squared and pWIN R-squared. In the gains domain, we found no correlation between risk premium and the amount of rEV information used [*r*_(89)_ = −0.14, *p* = 0.18] or the amount of pWIN information used [*r*_(89)_ = 0.04, *p* = 0.70]. In the losses domain, we found significant correlations between the risk premium metric and both the amount of rEV information used [*r*_(97)_ = −0.44, *p* < 0.0001] and the amount of pWIN information used [*r*_(97)_ = 0.25, *p* = 0.014]. Comparing these correlations across domains, the correlation between risk premium and the amount of rEV information used was significantly stronger in the losses domain (*z* = 2.25, *p* = 0.025), while there was no significant change in the strength of the correlations between risk premium and amount of pWIN information used across domains (*z* = 1.42, *p* = 0.16).

These results indicated that, in the losses domain participants with lower risk premium values made greater use of rEV information (maximizing) and relied less on pWIN information (satisficing). Interestingly, the zero-point for the risk premium metric reflects risk neutrality, so these results indicate that, for losses, the more an individual relied upon the maximizing information the more risk neutral their risk preferences were. Of note, this relationship is not a mathematical dependency (or by-product) of the task design or analyses (it is analytically possible for two people to have the same risk preference value yet employ different choice strategies).

This relationship between risk neutrality and maximizing strategies replicates a recent study using a blocked version of these tasks (gains trials first, then losses trials; Kurnianingsih et al., 2015). Curiously, in that study, these effects were found only for older adults in the losses domain, while younger adults showed no such relationship. Potential explanations for the current expansion of this relationship to younger adults include sampling differences or an interaction with the interleaved gains and losses trials.

### Relationship between absolute risk premium and choice strategies

We were curious about the domain-specificity of the relationship between risk premium and strategy, present for losses, and absent for gains. We note that there is a wider distribution of risk preferences in the gains domain, with the majority of people risk averse but a significant number of people who are risk seeking. In addition, there is a non-linearity in the risk premium metric as you cross zero. To test whether this range of values was obscuring the same relationship as found in the losses domain, we sought to transform the data to reflect the distance of each participant from risk neutrality (regardless of sign). Standardly, this would be accomplished by taking the absolute value, however with the nonlinear relationship across zero for risk premium, the conversion of negative risk premium values to positive requires the formula [absolute premium value = 1/(premium value +1) −1]. We performed this transformation for both the gains and losses domains, and re-ran the correlations between absolute risk premium and strategy. This resulted in significant negative correlations in both the gains [*r*_(89)_ = −0.30, *p* = 0.004] and losses [*r*_(97)_ = −0.36, *p* < 0.001] domains, with no significant difference across domains (*z* = 0.43, *p* = 0.67). The more each participant relied upon the maximizing rEV information, the more risk neutral their preferences were (Figures 5C,D), across both gains and losses domains.

### Relationship between numeracy and economic measures

We examined whether risk preferences and choice strategies were correlated with individual's numeracy ability. We found no significant correlations between risk premiums and numeracy score in the gains [*r*_(89)_ = 0.11, *p* = 0.28] or losses domains [*r*_(97)_ = −0.16, *p* = 0.11], concurring with Tymula et al. (2013), but contrary to the recent findings by Schley and Peters (2014). We also examined the relationship between numeracy and the reliance on rEV information. We found a positive correlation between the amount of rEV information used and numeracy score in both the gains [*r*_(102)_ = 0.27, *p* = 0.005] and losses domains [*r*_(102)_ = 0.23, *p* = 0.020], indicating that individuals with better numeracy abilities make more use of the calculable rEV information.

### Relationship between impulsivity and economic measures

We also examined whether risk preferences and choice strategies were correlated with individual's level of impulsivity. The average BIS-11 impulsiveness score across subjects was 63.62 (*SD* = 8.29). There was no significant correlations between risk premiums and impulsivity score in the gains [*r*_(89)_ = 0.04, *p* = 0.70] or losses domains [*r*_(97)_ = −0.03, *p* = 0.80], agreeing with Huettel et al. (2006). For choice strategy, we also looked at the relationship between impulsivity and the reliance on trial information, rEV and pWIN. There was also no significant correlations between the amount of rEV information used and impulsivity score in the gains [*r*_(102)_ = −0.13, *p* = 0.18] or losses domain [*r*_(102)_ = −0.05, *p* = 0.60], nor were there any significant correlations between the amount of pWIN information used and impulsivity in the gains [*r*_(102)_ = 0.14, *p* = 0.15] or losses domain [*r*_(102)_ = 0.05, *p* = 0.60]. These results indicate that the behavioral impulsivity measured by BIS-11 is independent from risk preference and choice strategy across both gains and losses.

## Discussion

We investigated the differences between gains and losses decision making by examining the interrelationships between risk preferences and choice strategies across the gains and losses domains. On average, we find that participants were risk averse for gains and risk neutral for losses. In opposition to the reflection effect, individual risk preferences were uncorrelated across the gains and losses domains, though cross-domain risk preferences were still able to predict choices better than chance or random preference. Investigating the strategies that individuals employ, individuals showed greater reliance on the maximizing strategy in the losses domain than in the gains domain. Interestingly, we identified a correlation between risk preferences and choice strategies in the losses domain in which the more individuals relied upon the maximizing strategy the more risk neutral their risk preferences were.

### Testing the sample reflection effect–average risk preferences in the gains and losses domains

First, we sought to replicate the classic pattern of risk preferences predicted by prospect theory (Kahneman and Tversky, 1979)—on average risk averse for gains and risk seeking for losses. In the gains domain, individuals were on average risk averse (based on risk premium and power function), concurring with prospect theory. In the losses domain, however, participants were on average risk neutral (risk premium metric) or weakly risk seeking (power function metric). These findings overall concur with the findings by Kahneman and Tversky (1979), indicating a sample-level reflection effect. We note, that this risk neutrality/weak risk seeking for losses still concurs with the original data that individuals are more willing to engage in gambles to prevent losses than to achieve gains—i.e., individuals are relatively more risk averse for gains than for losses.

### Testing the individual reflection effect–are individual preferences correlated across domains?

The reflection effect suggests that the individuals who are most risk averse in gains will be the most risk seeking for losses—is this true? No. We do not find a significant relationship in the direction predicted by the reflection effect, using either risk preference metric (premium or power function). Our results indicate that risk preferences for gains, cannot predict individual risk preferences for losses, in concurrence with prior research (Cohen et al., 1987; Schoemaker, 1990; Laury and Holt, 2000; Kurnianingsih et al., 2015; Mullette-Gillman et al., 2015a,b).

Interestingly, we find a non-significant correlation in the opposite direction of effect predicted by the reflection effect, using the risk premium metric (Table 1), in the same direction reported recently by Tymula et al. (2013). At the extreme, if this trend were to hold it would suggest that the individuals who were most risk averse for gains were not the most risk seeking for losses (as predicted by the reflection effect), but remained the most risk averse in losses.

The reflection effect was originally identified in the comparison of average group risk behavior across gains and losses (Kahneman and Tversky, 1979). It is possible to reconcile the presence of a sample-level reflection effect, with our correlations that are opposite the predicted direction. Simply, these two results suggest that, for a significant number of individuals, the difference between gains and losses risk preferences is a shift in preferences (an additive component, as indicated by our regression analysis for the power function metric). In other words, for many people, those who are most risk averse for gains shift to become less risk averse, while those that are least risk averse shift to become less risk averse, risk neutral, or even risk seeking (depending on the degree of the shift).

Alternatively, while it is common to discuss risk preference as a unitary stable concept (such as a personality trait), there is also evidence that risk preferences may be independent across different domains. For example, evidence suggests independence of risk attitudes across domains such as investment, insurance, health, recreational, work, and social decisions (Hershey and Schoemaker, 1980; Weber et al., 2002). Interestingly, though we did not find any correlation between risk preferences across domains, we found that individual's cross-domain risk preferences did contain information that was able to facilitate prediction of choice behavior. Therefore, although risk preferences may be strongly context and domain dependent, an underlying general risk preference may moderate cross-domain factors.

One interesting question raised by these results is how to consider mixed gambles (those with both possible gains and losses components). It is unclear whether gains and losses risk preferences would be predictive of behavior over mixed-gambles.

### Difference in choice strategies between the gains and losses domains

We examined whether the trial information individuals rely upon to make their choices differs across the gains and losses domains. While both domains featured greater reliance on the rEV information over the pWIN, there was even greater reliance on rEV information in the losses domain than in the gains domain. There is inherently no difference in the difficulty of calculations across the gains and losses domains, suggesting this result must be due to enhanced motivation in the losses domain; that participants were more willing to engage in the effortful rEV calculations to avoid possible losses than to reach possible gains. This concurs with recent studies that have found that incentives framed as losses result in higher work productivity compared to incentives framed as gains (Fryer et al., 2012; Hossain and List, 2012).

### Relationship between risk preference and choice strategy

In both the gains and losses domains, we found a negative correlation between distance from risk neutrality and the degree to which participants used the rEV information (the maximizing strategy in our task). Importantly, this relationship is not due to a mathematical dependency in the task or analyses, but is the result of the behavior of the participants (in other words, in our task/analyses it is possible for participants to have any pairing of risk preferences with any strategy value).

Risk neutral preferences suggest the absence of value modulation due to uncertainty—that participants were unbiased by uncertainty. As risk neutrality is the point where utility maximization converges with value maximization, this relationship indicates that those people who relied more on the use of rEV information, used the information not only to maximize their utility but were simultaneously maximizing the expected value of the outcomes.

In our task, the maximizing strategy requires deliberative cognitive processing, and we find a relationship between higher reliance on such deliberative reasoning and risk neutral preferences. In contrast, higher reliance on automatic cognitive processes bias preferences away from neutrality. These results concur with suggestions that risk may modulate decision making away from neutrality due to inclusion of affective responses (e.g., anticipated fear of loss; Loewenstein et al., 2001). Such differential influences of cognitive and affective processes is described by dual cognitive theory, in which there is a competition between slow-deliberative and automatic-effortless processing during the decision making process (Kahneman and Frederick, 2002; Evans, 2003). As a result, as individuals increased reliance on the maximizing strategy, this would have competitively reduced inclusion of affective biases, resulting in more neutral risk preferences.

An interesting possibility is that this relationship between risk preferences and choice strategy may provide a potential explanation as to why risk preferences appear to vary across different contexts (such as financial, health, and social domains; Weber et al., 2002). If risk preferences are driven by the choice strategy, and if the available types or quality of information varies across contexts, then a stable underlying risk preference could be differentially expressed across different contexts/domains. Similar choice strategies may reflect the same underlying cognitive processing modulating the risk preferences used to govern choices made. As such, varying risk preferences across contexts may be due to necessarily differential strategies due to domain-specific information.

### Explanation for framing effect?

These findings offer a potential explanation for the framing effect. Standardly, studies examining the framing effect observe a shift in risk preferences between the presented relative gains and relative losses, with participants more willing to accept gambles in which they can avoid a possible loss (for review, see Kühberger, 1998; Levin et al., 1998). We show that this shift in risk preference is not due to the reflection effect, which would have suggested that the same cognitive/neural processes are engaged in either domain. Rather, we see independence between risk preferences across the gains and losses domains, which suggests that different cognitive/neural mechanisms are engaged when prospects are framed as gains and losses and that an individual's choices in one domain cannot predict choices in the other domain. In other words, while the relative gain option and the relative loss option are mathematically equivalent there may be significant differences in the cognitive/neural systems that are engaged to process these options. If so, a simple transformation of the available options (such as altering the values from absolute to relative a mid-value) could result in dramatically different decisions as different cognitive processes/neural substrates are engaged.

## Conclusions

In this study, we found multiple indicators of independence and differentiation in gains and losses decision making. For risk preferences, utilizing two different preference metrics, we replicated the differentiation of average preferences for gains and losses, with risk averse for gains and risk neutral/seeking for losses. However, moving to individual participants, we were unable to show the interrelationship of risk preferences predicted by prospect theory and the reflection effect. Examining the strategies that participants employed to make their choices, across domains, individuals placed greater reliance on the effortful maximizing strategy, and the more they relied on this choice strategy the more their preferences were neutral. However, we did not show pure independence across gains and losses decision making. Cross-domain risk preferences still had predictive information about choices, even though we found not only non-significant but also opposite-signed correlations from that predicted by prospect theory's reflection effect.

Taken together, these findings suggest that gains and losses decision making are the result of both separable and overlapping cognitive/neural mechanisms. The separability is suggested by the independence of risk preferences across domains—even in intermixed gains and losses trials preferences across domains were uncorrelated. The overlap is suggested by the maintained predictive power of cross-domain risk preferences and the cross-domain relationship between risk preferences and strategies.

A possible explanation for such simultaneous separability and overlap is in the encoding and interactions of the valuative and executive processes. Although gains and losses are trivially related mathematically, and both provide motivation for decision making, they are the result of extremely different evolutionary pressures and the cognitive processes/neural mechanisms will reflect such convergence and divergence. Separabilities in behavior will arise to the degree there is differential/divergent neural encoding of the valuative/affective signals for gains and losses (Bartra et al., 2013; Pessiglione and Delgado, 2015). Overlapping behavior will arise from engagement of shared/convergent non-valuative processes, such as executive processes related to working memory and contingency processing (Mullette-Gillman and Huettel, 2009; Miller and Cohen, 2001). As these shared executive and differential valuative processes interact (Mullette-Gillman et al., 2011), they will result in aspects of behavior that exhibit both convergence and divergence between the gains and losses domains.

## Author contributions

YA and OM designed the experiment, analyzed the data and wrote the manuscript. YA collected the data.

### Conflict of interest statement

The authors declare that the research was conducted in the absence of any commercial or financial relationships that could be construed as a potential conflict of interest.
